# The Relative Importance of Innate Immune Priming in *Wolbachia*-Mediated Dengue Interference

**DOI:** 10.1371/journal.ppat.1002548

**Published:** 2012-02-23

**Authors:** Edwige Rancès, Yixin H. Ye, Megan Woolfit, Elizabeth A. McGraw, Scott L. O'Neill

**Affiliations:** School of Biological Sciences, Monash University, Clayton, Australia; Stanford University, United States of America

## Abstract

The non-virulent *Wolbachia* strain *w*Mel and the life-shortening strain *w*MelPop-CLA, both originally from *Drosophila melanogaster*, have been stably introduced into the mosquito vector of dengue fever, *Aedes aegypti*. Each of these *Wolbachia* strains interferes with viral pathogenicity and/or dissemination in both their natural *Drosophila* host and in their new mosquito host, and it has been suggested that this virus interference may be due to host immune priming by *Wolbachia*. In order to identify aspects of the mosquito immune response that might underpin virus interference, we used whole-genome microarrays to analyse the transcriptional response of *A. aegypti* to the *w*Mel and *w*MelPop-CLA *Wolbachia* strains. While *w*Mel affected the transcription of far fewer host genes than *w*MelPop-CLA, both strains activated the expression of some immune genes including anti-microbial peptides, Toll pathway genes and genes involved in melanization. Because the induction of these immune genes might be associated with the very recent introduction of *Wolbachia* into the mosquito, we also examined the same *Wolbachia* strains in their original host *D. melanogaster*. First we demonstrated that when dengue viruses were injected into *D. melanogaster*, virus accumulation was significantly reduced in the presence of *Wolbachia*, just as in *A. aegypti*. Second, when we carried out transcriptional analyses of the same immune genes up-regulated in the new heterologous mosquito host in response to *Wolbachia* we found no over-expression of these genes in *D. melanogaster*, infected with either *w*Mel or *w*MelPop. These results reinforce the idea that the fundamental mechanism involved in viral interference in *Drosophila* and *Aedes* is not dependent on the up-regulation of the immune effectors examined, although it cannot be excluded that immune priming in the heterologous mosquito host might enhance the virus interference trait.

## Author Summary

*Wolbachia pipientis* is an inherited intracellular bacterium that is widespread in insects. Because of its ability to interfere with various pathogens such as dengue viruses, nematodes and *Plasmodium* in insects, it has been proposed as a possible tool to control insect-transmitted disease. Recently, two strains of *Wolbachia* that interfere with RNA viruses in their natural host, *Drosophila melanogaster*, were introduced into the naturally uninfected mosquito vector of dengue fever, *Aedes aegypti*. As in their natural host, those two strains block the replication and the dissemination of viruses in the mosquito. Some studies suggest that pathogen blocking is due to *Wolbachia* priming the insect innate immune system. Here, we show that *Wolbachia* induces transcription of some immunity related genes only in its new host *A. aegypti*, and not in its natural host *D. melanogaster*, while *Wolbachia* reduces dengue replication in both hosts. These results suggest that immune priming by *Wolbachia* might not be the only mechanism responsible for viral interference.

## Introduction

*Wolbachia* is a vertically transmitted endosymbiont that infects up to 70% of all insect species. The association is usually not obligatory for the insect and many *Wolbachia* strains assure their maintenance in populations by manipulating the reproduction of their host [1]. Interestingly, some strains interfere only weakly with host reproduction but still spread and are maintained in insect populations [2]. Their success may be explained by an additional positive selective advantage associated with *Wolbachia* infection. One possible advantage is the recently described pathogen blocking that the bacterium confers upon its host. This phenotype was first demonstrated in *Drosophila*, where *Wolbachia* induces resistance to different types of RNA viruses by reducing viral titer and/or making the host resistant to virus pathogenicity [3]–[5]. The extent and nature of blocking vary according to the virus and the *Wolbachia* strains tested. For example, *Wolbachia* reduces the titer of the closely related DCV and Nora viruses in *Drosophila melanogaster* and *D. simulans* [4], [5] and as a consequence, the pathology associated with those two viruses is less intense in *Wolbachia*-infected flies [3]–[5]. In contrast, the bacterium does not affect FHV titer in *Drosophila* but still reduces the pathogenicity of the virus [3]–[5]. In *D. simulans*, the *w*Au *Wolbachia* strain has a strong effect against DCV pathogenicity, whereas the strains *w*Ha and *w*No do not [5]. This observation is thought to be related to the low infection density of *w*Ha and *w*No in *Drosophila* compared to that of the *w*Au strain [5].

*Wolbachia* does not naturally infect the main mosquito vector of dengue viruses, *Aedes aegypti*. However, two *Wolbachia* strains originally isolated from *D. melanogaster* (*w*MelPop-CLA and *w*Mel) and one strain originally from *A. albopictus* (*w*AlbB) have been successfully trans-infected into *A. aegypti* and subsequently stably maintained [6]–[8]. All of these strains express cytoplasmic incompatibility in *A. aegypti* as they do in their original hosts, *D. melanogaster* and *A. albopictus* [6]–[8]. In addition, the virulent *w*MelPop-CLA strain that lacks normal replication control and reduces lifespan in *D. melanogaster* also does so in *A. aegypti* [7]. As observed in *Drosophila*, *Wolbachia*-infected *A. aegypti* are more resistant to RNA virus infection, including dengue and chikungunya [8], [9], as well as bacteria, nematodes and *Plasmodium* [9], [10]. Transient somatic infection of the main African vector of human malaria, *Anopheles gambiae*, by *w*MelPop also significantly decreased *Plasmodium* infection intensity [11].

The molecular mechanisms involved in *Wolbachia*-mediated pathogen protection are still not clear. One plausible hypothesis is that *Wolbachia* interferes with pathogens by pre-activating the immune response of its host. The virulent strain *w*MelPop-CLA activates a wide range of immune processes in *A. aegypti*, including the Toll and Imd signaling pathways, anti-microbial peptide synthesis, melanization, RNA interference and opsonisation [9], [10] and the somatic infection of *An. gambiae* by *w*MelPop caused an increase in expression of opsonisation genes [11]. Evidence for the role of opsonisation in protection against *Plasmodium* in this host was demonstrated by knocking down expression of the TEP1 gene [11]. Transcriptional analyses of *A. aegypti* immunity genes showed that *w*AlbB increases expression of genes in the Toll pathway and in particular the anti-microbial peptide gene, defensin [12]. Activation of the Toll pathway has been shown previously to suppress dengue infection in mosquitoes [13]. Each of these previous studies was limited in that they examined *Wolbachia* strains that were either virulent and/or recently introduced into naturally uninfected host species. To our knowledge, only two previous studies have examined expression of innate immune genes in insect species naturally infected by *Wolbachia*, including *D. simulans*, *D. melanogaster* and *A. albopictus*. In these cases no differences in gene regulation were observed between *Wolbachia*-infected insects and their uninfected counterparts [14], [15].

Since all previous studies that have shown evidence of immune activation have been based on recently established heterologous infections, it is unclear how generalizable the *Wolbachia* activation of the mosquito immune system is for all insects. To determine whether immune up-regulation by the bacterium is a general mechanism underlying *Wolbachia*-induced dengue interference, we performed transcriptional analyses on the two heterologous associations, *w*Mel and *w*MelPop-CLA infected *A. aegypti*, and the two native associations, *w*Mel and *w*MelPop infected *D. melanogaster*. We also tested if the non-virulent strain *w*Mel blocks dengue replication in *Drosophila* as it does in mosquitoes. If the same strain of *Wolbachia* blocks the replication of the same virus in different hosts, we can make the parsimonious assumption that virus interference is likely to have a common mechanistic basis across different hosts. This cross-comparison with the two *Wolbachia* strains and dengue virus in both native and heterologous hosts allows us to remove extraneous effects, such as recent transfer to a heterologous host or virulence associated with the *w*MelPop infection, that might confound an understanding of the underlying mechanistic basis of *Wolbachia*-induced viral interference.

This study also contributes to our understanding of the physiological impact of *w*Mel infection on *A. aegypti*. This is of particular relevance because *w*Mel-infected *A. aegypti* have been released in north Queensland, Australia, in a field trial using *Wolbachia* as a biocontrol mechanism for dengue [16]. In the near future, this biological tool is also likely to be applied in dengue-endemic areas of Vietnam and Indonesia [17].

## Results

### Transcriptional response of *Aedes aegypti* to *Wolbachia* infection

We examined the global transcriptional response of mosquitoes to *Wolbachia* infection using microarrays. We compared the responses of 8 day old, non blood-fed *A. aegypti* females stably transinfected with *w*MelPop-CLA (line PGYP1) or *w*Mel (line MGYP2) to those of the corresponding tetracycline-cured lines PGYP1.tet and MGYP2.tet. The design of the microarray included 12,336 transcripts, which represented 12,270 of the 15,988 genes present in the *A. aegypti* genome. We considered a gene to be up- or down-regulated by *w*MelPop-CLA or *w*Mel infection if the fold change in transcription relative to non-infected mosquitoes was significantly different from 1.0 and greater than 1.5. Because the *Drosophila* genome is better characterized, we identified *Drosophila* orthologs of each *A. aegypti* gene where possible to obtain additional functional annotations.

**Figure 1:**
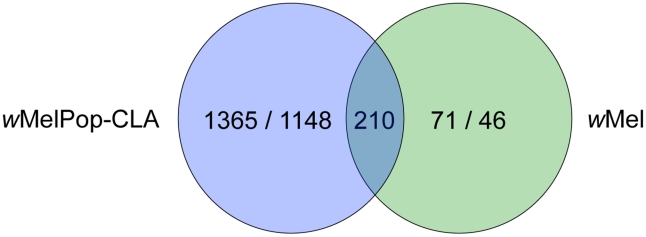
Venn diagram showing significant expression change in response to infection in *A. aegypti* infected with *w*MelPop-CLA or *w*Mel. The overlap region corresponds to *A. aegypti* gene transcripts significantly up- and down-regulated in response to both strains. Numbers indicate gene transcripts up-regulated/gene transcripts down-regulated.

The *w*MelPop-CLA infection affected the transcription of far more genes (2723) than the *w*Mel infection (327) (Figure 1). This is likely related to *w*MelPop-CLA's higher density in its host, broader cellular tropism and pathogenicity [8], [9], [18]. Based on Gene Ontology (GO) annotations, *w*MelPop-CLA has an impact on a broader range of *A. aegypti* biological and molecular functions than *w*Mel (Table 1, 2).

**Table 1:** Gene Ontology (GO) terms over-represented among gene transcripts significantly up-regulated in *w*Mel-infected *A. aegypti*.

GO ID	Term description	Adjusted *P*-values
**Biological process**		
GO:0009607	Response to biotic stimulus	2.16E-05
GO:0006508	Proteolysis	1.05E-04
GO:0051704	Multi-organism process	2.33E-04
GO:0019538	Protein metabolic process	1.09E-02
GO:0006952	Defense response	1.27E-02
**Molecular function**		
GO:0017171	Serine hydrolase activity	2.16E-05
GO:0008233	Peptidase activity	2.81E-03
GO:0004175	Endopeptidase activity	6.40E-03
GO:0003824	Catalytic activity	6.89E-03
GO:0005529	Iron ion binding	2.53E-02
GO:0016787	Hydrolase activity	3.65E-02
**Cellular component**		
GO:0005576	Extracellular region	4.85E-04

Adjusted *P*-values are the *P*-values generated by the Ontologizer program [38], using the Benjamini-Hochberg method.

**Table 2:** Gene Ontology (GO) terms over-represented among gene transcripts significantly up-regulated in *w*MelPop-CLA-infected *A. aegypti*.

GO ID	Term description	Adjusted *P*-values
**Biological process**		
GO:0006508	Proteolysis	5.87E-15
GO:0009308	Amine metabolic process	9.22E-08
GO:005114	Oxidation reduction	4.97E-07
GO:0005975	Carbohydrate metabolic process	7.41E-05
GO:0009607	Response to biotic stimulus	2.16E-04
GO:0055085	Transmembrane transport	8.08E-04
GO:0044271	Cellular nitrogen compound biosynthetic process	2.72E-03
GO:0006952	Defense response	3.19E-03
GO:0022610	Biological adhesion	3.82E-03
GO:0051704	Multi-organism process	7.77E-03
GO:0051604	Protein maturation	9.19E-03
GO:0019538	Protein metabolic process	1.18E-02
GO:0002376	Immune system process	1.87E-02
GO:0043565	Chemical homeostasis	2.27E-02
GO:0051179	Localization	3.08E-02
GO:0071554	Cell wall organization or biogenesis	3.50E-02
GO:0044283	Small molecule biosynthetic process	4.96E-02
GO:0010876	Lipid localization	5.00E-02
**Molecular function**		
GO:0005506	Iron ion binding	3.98E-16
GO:0003824	Catalytic activity	6.62E-10
GO:0046906	Tetrapyrrole binding	1.31E-09
GO:0005215	Transporter activity	1.27E-06
GO:0030246	Carbohydrate binding	4.29E-06
GO:0009055	Electron carrier activity	6.98E-06
GO:0004857	Enzyme inhibitor activity	3.31E-05
GO:00164901	Oxidoreductase activity	4.12E-05
GO:0008233	Peptidase activity	7.56E-05
GO:0017171	Serine hydrolase activity	1.08E-04
GO:0061134	Peptidase regulator activity	2.23E-04
GO:0005509	Calcium ion binding	3.99E-04
GO:0005102	Receptor binding	3.82E-03
GO:0005044	Scavenger receptor activity	4.70E-03
GO:0005515	Protein binding	5.05E-03
GO:0004047	Aminomethyltransferase activity	1.08E-02
GO:0043565	Sequence-specific DNA binding	1.64E-02
**Cellular component**		
GO:0016020	Membrane	5.79E-16
GO:0005576	Extracellular region	4.32E-09
GO:0043234	Protein complex	4.62E-03
GO:0005856	Cytoskeleton	7.77E-03

Adjusted *P*-values are the *P*-values generated by the Ontologizer program [38], using the Benjamini-Hochberg method.

Many of the changes in gene regulation observed in mosquitoes infected with the virulent strain *w*MelPop-CLA are likely to be responses to the high physiological cost imposed by that strain. To identify mechanisms more likely to be involved in pathogen interference, we decided to focus on the 210 gene transcripts that showed significant changes in expression in both PGYP1 and MGYP2 compared to uninfected mosquitoes (Figure 1). Among those genes, 138 gene transcripts had functional annotations (Table S1).

Most of the 210 transcripts were either up-regulated in both PGYP1 and MGYP2 or down-regulated in both lines (Table S1). However, the magnitude of response was typically greater to *w*MelPop-CLA infection (Table S1). One of the few genes differentially expressed between PGYP1 and MGYP2 is AAEL002487, which is up-regulated in MGYP2 and down-regulated in PGYP1. This gene encodes the protein P53 regulated pa26 nuclear protein sestrin (dSesn in *Drosophila*) (Table S1). This protein is involved in the regulation of the target of rapamycin (TOR), a key protein in age-related pathologies like life-shortening or muscle degeneration [19], two phenotypes exclusively associated with *w*MelPop-CLA pathogenicity in *A. aegypti* [7], [20]. Among the 210 genes, most of the genes showing the greatest up-regulation are immune genes (Table S1). Gene Ontology (GO) annotations also revealed enrichment in genes related to immunity and proteolysis for MGYP2 and PGYP1 (Table 1, 2). The results obtained for PGYP1 are in accordance with a previous study of *A. aegypti* infected by *w*MelPop-CLA [10].

### Common immune pathways activated by *w*MelPop-CLA and *w*Mel in *A. aegypti*

The virulent strain *w*MelPop-CLA significantly affected regulation of many characterized immune genes in the mosquito (Table S2, [10]). By comparison, many fewer of these genes were activated by *w*Mel (Table 3, S1, S3). Those included genes encoding anti-microbial peptides, four cecropins (CECE, CECF, CECN, CECD), one defensin (DEFC) and one diptericin (DPT1). The magnitude of change in expression was substantial for some of these genes. The activation of these peptides is regulated by both Toll and Imd pathways, but we found up-regulation only of some Toll pathway genes, including the peptidoglycan recognition protein PGRP-SA and the Gram-negative binding proteins GNBPB4 and GNBPA1 (GNBP1 *Drosophila* homologs, Table 3). The Toll pathway effector defensin was the most highly up-regulated immune gene in *A. aegypti* infected by *w*Mel (Table 3). This is consistent with the results of Bian et al [12], who examined immune gene expression in heterologous *w*AlbB infection in *A. aegypti* and found that among the immune genes tested defensin was also the most up-regulated.

**Table 3:** *A. aegypti* putative immune transcripts significantly up-regulated in response to both *w*MelPop-CLA and *w*Mel infections.

	*w*MelPop-CLA		*w*Mel				
Transcripts ID	AFC	q-value	AFC	q-value	Description	Dm Gene ID H	Dm Symbol
**Anti-microbial peptides**							
AAEL000598-RA	10.44	1.83E-04	2.93	4.00E-03	cecropin (CECD)	no homolog	
AAEL000611-RA	125.52	9.63E-06	12.62	6.41E-03	cecropin (CECE)	no homolog	
AAEL000625-RA	53.83	3.65E-05	6.07	9.84E-03	cecropin (CECF)	no homolog	
AAEL000621-RA	47.31	1.14E-05	10.11	4.10E-03	cecropin (CECN)	no homolog	
AAEL003832-RA	70.76	7.09E-06	22.99	2.89E-03	defensin-C (DEFC)	FBgn0010385	Def
AAEL004833-RA	2.72	6.72E-05	1.53	5.46E-03	diptericin 1 (DPT1)	no homolog	
**Toll pathway**							
AAEL007993-RA	9.33	7.09E-06	1.90	4.81E-03	clip-domain serine protease (CLIPB27)	FBgn0039494	grass
AAEL007626-RA	3.04	2.68E-05	1.67	9.05E-03	gram-negative binding protein (GNBPA1)	FBgn0040323	GNBP1
AAEL009178-RA	3.72	8.98E-04	7.50	6.19E-03	gram-negative binding protein (GNBPB4)	FBgn0040323	GNBP1
AAEL011624-RA	2.55	4.84E-04	2.00	7.53E-03	granzyme A precursor	FBgn0003450	snk
AAEL009474-RA	6.76	5.10E-05	2.96	5.69E-03	peptidoglycan recognition protein (PGRPS1)	FBgn0030310	PGRP-SA
AAEL010867-RA	4.27	1.15E-04	1.76	4.59E-03	serine protease	FBgn0003450	snk
**Melanization**							
AAEL000024-RA	2.18	1.72E-04	1.54	9.33E-03	dopachrome-conversion enzyme (DCE)	FBgn0041710	yellow-f
AAEL013501-RA	32.84	2.53E-05	2.71	4.81E-03	pro-phenoloxidase (PPO4)	FBgn0000165	
AAEL003642-RA	8.29	7.09E-06	3.46	1.91E-03	serine protease	FBgn0037515	Sp7
AAEL013936-RA	1.65	6.22E-04	1.56	3.52E-03	serine protease inhibitor (SRPN4)	FBgn0031973	Spn28D
**Other putative immune related genes**							
AAEL005641-RA	31.47	3.97E-05	5.27	2.68E-03	C-type lectin - galactose binding (CTLGA5)	no homolog	
AAEL011621-RA	5.84	2.50E-04	2.35	2.89E-03	C-type lectin - mannose binding (CTLMA13)	no homolog	
AAEL011453-RA	4.15	3.79E-05	1.89	8.54E-03	C-type lectin (CTL14)	FBgn0053533	lectin-37Db
AAEL011408-RA	3.06	2.16E-05	1.99	5.26E-04	C-type lectin (CTL21)	no homolog	
AAEL002524-RA	7.38	1.20E-04	4.10	9.78E-03	C-type lectin (CTL24)	no homolog	
AAEL002601-RA	7.31	6.12E-05	2.31	2.33E-03	clip-domain serine protease (CLIPA1)	FBgn0033321	CG8738
AAEL014349-RA	6.74	7.09E-06	2.04	3.49E-03	clip-domain serine protease (CLIPB15)	no homolog	
AAEL000059-RA	2.10	4.14E-04	1.68	8.98E-03	clip-domain serine protease (CLIPB19)	no homolog	
AAEL001084-RA	16.39	7.09E-06	4.25	3.80E-03	clip-domain serine protease (CLIPB21)	no homolog	
AAEL008668-RA	4.53	6.51E-05	2.00	7.73E-03	clip-domain serine protease (CLIPB22)	no homolog	
AAEL006674-RA	1.85	2.22E-04	1.53	4.82E-03	clip-domain serine protease (CLIPB29)	no homolog	
AAEL000099-RA	4.26	2.27E-05	2.11	2.83E-03	clip-domain serine protease (CLIPB33)	no homolog	
AAEL005431-RA	22.66	1.85E-05	3.95	2.76E-03	clip-domain serine protease (CLIPB37)	no homolog	
AAEL005093-RA	11.58	3.13E-05	3.05	5.87E-03	clip-domain serine protease (CLIPB46)	no homolog	
AAEL010773-RA	3.64	4.41E-05	2.45	1.50E-03	clip-domain serine protease (CLIPE10)	no homolog	
AAEL001098-RA	5.01	6.49E-05	2.00	7.95E-03	clip-domain serine protease, putative	no homolog	
AAEL009861-RB	2.20	1.37E-04	2.06	8.98E-03	conserved hypothetical protein	FBgn0034638	CG10433
AAEL009861-RD	2.20	6.66E-05	2.08	7.11E-03	conserved hypothetical protein	FBgn0034638	CG10433
AAEL009861-RC	2.02	2.50E-04	1.66	7.11E-03	conserved hypothetical protein	FBgn0034638	CG10433
AAEL008473-RA	10.52	6.16E-03	1.91	3.35E-05	cysteine-rich venom protein, putative	FBgn0031412	CG16995
AAEL000374-RA	15.30	8.75E-03	2.15	3.10E-05	cysteine-rich venom protein, putative	no homolog	
AAEL012956-RA	3.81	1.11E-04	2.39	4.68E-03	elastase, putative	no homolog	
AAEL002022-RA	5.15	3.40E-04	2.65	3.20E-03	protein serine/threonine kinase, putative	FBgn0011695	PebIII/phk2
AAEL001964-RA	4.45	6.57E-05	1.90	4.74E-03	protein serine/threonine kinase, putative	FBgn0011695	PebIII/phk2
AAEL002585-RA	8.05	2.19E-05	1.66	7.61E-03	serine protease	FBgn0028864	CG18477
AAEL002624-RA	6.65	3.16E-05	1.89	2.74E-03	serine protease	FBgn0028514	CG4793
AAEL002610-RA	6.93	1.14E-05	2.10	8.54E-03	serine protease	FBgn0032638	CG6639
AAEL002301-RA	3.85	2.75E-05	2.18	7.63E-03	serine protease	no homolog	
AAEL003697-RA	3.11	3.05E-05	1.77	6.42E-03	serine protease inhibitor (SRPN17)	no homolog	
AAEL006136-RA	4.83	3.30E-05	2.17	3.66E-03	serine protease, putative	FBgn0038211	CG9649
AAEL006434-RA	3.53	4.42E-05	1.80	8.62E-03	serine protease, putative	no homolog	
AAEL013033-RA	3.18	1.52E-05	2.32	5.22E-03	serine protease, putative	no homolog	
AAEL013432-RA	2.56	6.78E-05	3.84	3.31E-03	serine protease, putative	no homolog	
AAEL004761-RA	1.89	3.12E-04	1.67	3.93E-03	serine/threonine-protein kinase MAK	FBgn0051711	
AAEL015458-RA	55.38	7.09E-06	12.23	1.88E-05	transferrin	FBgn0022355	Tsf1

Transcripts are ranked by biological process and/or molecular function. Transcript identifiers (Transcript ID) and Description were compiled from Vectorbase. *D. melanogaster* Gene Identifier Homolog (Dm Gene ID H) and Dm Symbol were compiled from Flybase. AFC, Absolute Fold Change.

Excluding anti-microbial peptides and the Toll pathway, the only other immune response activated by both *w*Mel and *w*MelPop-CLA in *A. aegypti* was melanization. Four genes in this pathway were up-regulated: one pro-phenoloxidase (PPO4), one dopachrome-conversion enzyme (DCE) that converts dopachrome into 5,6-dihydroxyindole just before melanin production by phenoloxidase [21], one putative protease inducer sp7 and one protease inhibitor Srpn4 (Table 3). The activation of these genes suggests that production of melanin is induced in *Wolbachia*-infected mosquitoes.

### Effect of *Wolbachia* on dengue virus in *Drosophila*

Since a comparative approach between *Drosophila* and *Aedes* to examine the effect of immune activation on virus interference is predicated on an assumption that dengue virus interference also occurs in *Wolbachia*-infected *Drosophila*, we tested the ability of dengue virus serotype 2 (DENV-2) to grow in *Drosophila* carrying the *w*Mel *Wolbachia* strain.

**Figure 2:**
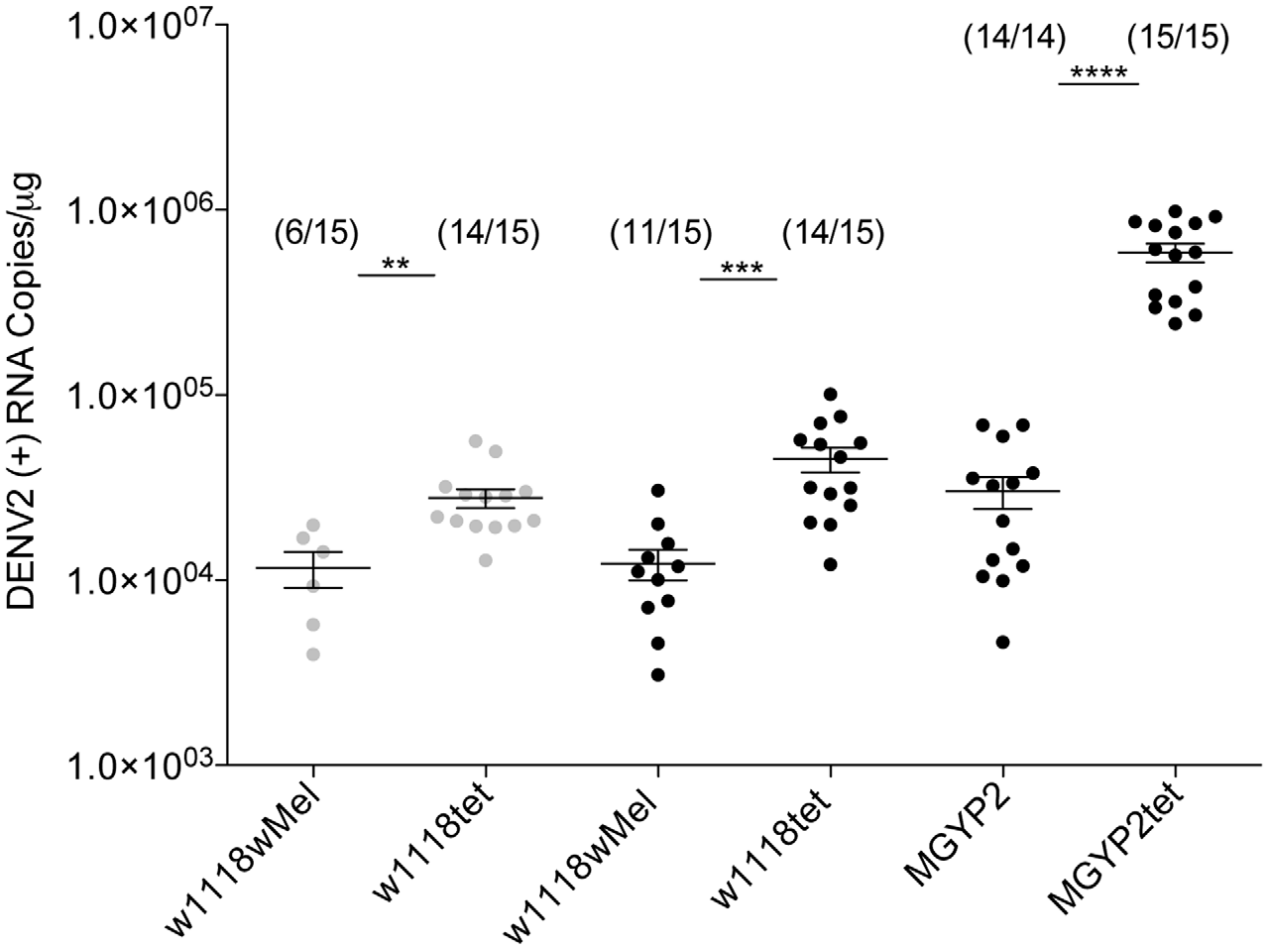
Dengue blocking in *D. melanogaster* and *A. aegypti* infected by *Wolbachia* strain *w*Mel. 69 µl of 10^7^ pfu/ml of DENV2 strain 92T (grey circles) and DENV2 strain ET300 (black circles) were injected into flies (*w^1118^w*Mel) and mosquitoes (MGYP2) infected by *w*Mel and their tetracycline-treated uninfected counterparts (*w^1118^*tet and MGYP2tet). Dengue levels in individual insects were determined 8 days post-infection by RT-PCR using a TaqMan assay specific to dengue in 1 µg of total RNA. The fraction of flies that had detectable dengue infections is shown above each set of data points. (n = 15, Mann-Whitney *U* test, **: p<0.01, ***:p<0.001, ****:p<0.0001).

For both dengue virus strains, 92T and ET300, the total number of flies infected by dengue was lower in the presence of *w*Mel, with only 40% of flies detected positive for the 92T strain compared with 93% for the *Wolbachia*-uninfected control. Similarly for the ET300 strain, 73% of *Wolbachia*-infected flies were positive for dengue compared to 93% for the *Wolbachia*-uninfected control (Figure 2). In addition, for the flies that did become infected with dengue the amount of DENV-2 RNA present was significantly reduced in the presence of *w*Mel (Figure 2). It was unsurprising to note that dengue grew to higher levels when injected into its natural mosquito host compared to *Drosophila* but regardless of absolute virus levels significant *Wolbachia* interference effects were detected in both insect species. Dengue injection in flies did not have an effect on insect life span nor increased mortality compared to controls (data not shown).

### Effect of *w*MelPop and *w*Mel on the *Drosophila melanogaster* immune system

Considering that the *Wolbachia* strains *w*MelPop [22] and *w*Mel in their original host interfere with natural *Drosophila* RNA viruses and also with dengue virus replication, we then investigated the possibility that both *Wolbachia* strains boost *Drosophila* immunity as seen in the heterologous mosquito host. We examined by quantitative real time PCR the expression of the *Drosophila* homologs of the mosquito immune genes identified through microarray analysis to be up-regulated in the presence of *Wolbachia*.

There have been multiple gene losses and gene duplications in immune gene families in both flies and mosquitoes [23], and we were therefore unable to reliably identify all orthologs for our anti-microbial peptide genes and pro-phenoloxidase genes of interest. Thus, we targeted all the cecropin, diptericin and pro-phenoloxidase genes present in the genome of *D. melanogaster*. In total 13 immune genes were analyzed: seven anti-microbial peptide genes, two Toll pathway genes and four melanization genes (Table 4).

**Table 4:** Immune transcript analyses in *D. melanogaster* infected with *w*MelPop and *w*Mel.

	*w*MelPop			*w*Mel				
Gene ID	AFC	q-value		AFC	q-value		Description	Symbol
**Anti-microbial peptides**								
FBgn0000276	2.24	0.030	*	−1.59	0.324		cecropin A1	CecA1
FBgn0000277	1.63	0.109		1.58	0.597		cecropin A2	CecA2
FBgn0000278	ND			ND			cecropin B	CecB
FBgn0000279	ND			ND			cecropin C	CecC
FBgn0004240	1.25	0.661		−1.16	0.743		diptericin	Dpt
FBgn0034407	1.37	0.661		−1.13	0.743		diptericin B	DptB
FBgn0010385	1.27	0.398		1.24	0.591		defensin	Def
**Toll pathway**								
FBgn0030310	−1.49	0.030	*	1.11	0.168		peptidoglycan recognition protein SA	PGRP-SA
FBgn0040323	1.05	0.631		1.29	0.002	**	gram-negative binding protein 1	GNBP1
**Melanization**								
FBgn0261363	−2.6	0.008	**	−1.69	0.142			CG42640
FBgn0261362	1.67	0.011	*	−1.47	0.030	*	pro-phenoloxidase A1	proPO-A1
FBgn0033367	1.04	0.743		−1.39	0.154			CG8193
FBgn0041710	1.08	0.631		−1.01	0.661		yellow-f	yellow-f
**Other**								
FBgn0022355	−2.25	0.008	**	−1.15	0.324		transferrin 1	Tsf1
FBgn0015221	−1.99	0.109		−1.22	0.661		ferritin 2 light chain homologue	Fer2lch

Transcripts are ranked by biological process and/or molecular function. Gene identifiers (Gene ID), Description and Symbol were compiled from Flybase. AFC, Absolute Fold Change, ND, No Detection. Asterisks indicate a statistically significant difference (n = 10, Mann-Whitney *U* test with q-value adjustment, *: q<0.05, **: q<0.01).

No significant changes in the expression of anti-microbial peptide genes were observed for *w* ^1118^ *w*MelPop or *w* ^1118^ *w*Mel, except for cecropin A1 (Table 4). The expression of cecropin A1 was two-fold higher in the presence of *w*MelPop, whereas no change was observed in the presence of *w*Mel (Table 4). No gene expression was detected for the cecropins B and C for either of the *Drosophila* lines tested. No significant changes in diptericin transcription were observed in *Wolbachia*-infected flies, which suggests that the Imd signaling pathway is not stimulated by *Wolbachia* in *Drosophila*. The expression patterns of two major genes in the Toll pathway, PGRP-SA and GNBP1, differed between flies infected by *w*Mel and *w*MelPop. A slight inhibition of PGRP-SA was observed in flies infected by *w*MelPop, while in *w*Mel-infected flies there was no effect. For GNBP1, a minor but significant difference, 1.29-fold change, was observed for *w^1118^w*Mel but not for *w^1118^w*MelPop (Table 4). The expression of only a single melanization gene was affected by *w*Mel infection: proPO-A1 was down-regulated. In contrast, in flies infected with *w*MelPop, proPO-A1 was significantly up-regulated and another melanization gene, CG42640, was down-regulated (Table 4).

An enrichment of gene transcripts encoding the iron binding proteins transferrin and ferritin was detected in the data obtained from the *A. aegypti* transcriptome analysis in response to *w*Mel and *w*MelPop-CLA infections (Table 1, 2, S1). These proteins have multiple functions in insects, including iron homeostasis and immunity [24], two potential mechanisms that could be involved in *Wolbachia*-mediated pathogen protection. The expression of the genes encoding transferrin 1 (Tsf1) and the light chain of ferritin (Fer2lch) was evaluated in *w^1118^w*Mel and *w^1118^w*MelPop compared to *w^1118^*tet. However, no induction was found in *Wolbachia*-infected flies (Table 4) and *w*MelPop infection even significantly reduced the expression of transferrin.

The expression of immune genes was also tested in the same fly lines (*w^1118^w*Mel and *w^1118^*tet) infected with DENV-2, strain 92T. Even in the presence of dengue, *w*Mel infection did not increase the expression of anti-microbial peptides and pro-phenoloxidases (Figure S1). No correlation was found between the amount of dengue detected and the level of expression for each of the anti-microbial peptide and pro-phenoloxidases genes tested in each fly line (Figure S2).

## Discussion

Host immune priming by *Wolbachia* offers an appealing mechanistic explanation for pathogen blocking as it is conceivable that this single effect could lead to protection against a diversity of pathogens. The objective of this study was to compare the effect of two closely-related strains of *Wolbachia* on the immune system of hosts where the age of the *Wolbachia* association differs. By comparing *w*MelPop-CLA and *w*Mel we could exclude any potential immune activation that may simply be due to the virulence of the *w*MelPop-CLA infection. By examining both *D. melanogaster* and *A. aegypti*, we were able to dissect aspects of the immune response that may be attributed solely to a host's response to a recently acquired *Wolbachia* infection. This analysis depends on an assumption that the mechanism of virus interference is similar in the two insect hosts. Considering that *Wolbachia* infection in *Drosophila* interferes with dengue replication, as it does in *A. aegypti*, the assumption of a similar mechanism seems parsimonious. Moreover the success of maintaining dengue in *Drosophila*, even if viral replication is not as strong as in *A. aegypti*, provides a tractable genetic model for future studies into the mechanistic basis of *Wolbachia*-mediated dengue interference.

A previous analysis of *A. aegypti* whole genome transcription in response to *w*MelPop-CLA revealed strong immune induction by the bacterium [10]. In this present study, a similar approach was taken to analyze the impact of the non-virulent *w*Mel strain on the immune system of *A. aegypti*, in comparison with the *w*MelPop-CLA strain. The results obtained revealed that *w*Mel induces the activation of far fewer immunity genes in the mosquito. The comparative analysis between the different lines identified common responses only for genes encoding anti-microbial peptides, the Toll pathway and melanization-associated proteins. Recent studies have provided important insights into *A. aegypti* immune response to dengue virus, showing that the Toll pathway and anti-microbial peptides are important for the mosquito's defense against dengue infection [13], [25]. Melanization is also a prominent immune response in insects against parasites like malaria and nematodes [26] but as far as we know it has never been demonstrated for dengue.

The main anti-viral pathway, RNA interference [27], seems to be activated exclusively by *w*MelPop-CLA. Several pieces of evidence also indicate that RNAi cannot explain virus blocking. First, Glaser et al [28] showed that even in Ago2 (a key gene in the RNAi pathway) mutant flies, *Wolbachia* infection increases resistance to viruses. Second, Frentiu et al [29] demonstrated that *w*MelPop-CLA induces complete inhibition of dengue virus replication in the C6/36 cell line that has been shown to be defective in the RNAi pathway [30].

This comparative analysis between *w*Mel and *w*MelPop-CLA infection within *A. aegypti* supports the potential implication of anti-microbial peptides and Toll pathway activation in dengue virus interference by the bacterium. If we assume that the fundamental mechanism involved in *Wolbachia*-mediated dengue interference is the same in mosquitoes and flies, and this mechanism is immune-based, then the same constitutive immune induction should also be observed in *D. melanogaster* infected by *w*Mel or *w*MelPop. We tested for transcriptional changes of the same immune genes identified through microarray analysis in *D. melanogaster* in response to *Wolbachia* infection, and identified a number of statistically significant changes. However, in no case were these changes consistent between *w*Mel and *w*MelPop infection. Furthermore, if we employed the same threshold for biological significance we used for our microarray data, that a gene is significantly up-regulated by *Wolbachia* infection only when its level is changed at least 1.5-fold compared with non-infected flies, we would conclude that *w*Mel did not constitutively prime any of the different immune genes tested in its natural host *D. melanogaster*. Those results are in accordance with previous data showing no pre-activation of different immune genes in *D. melanogaster*, *D. simulans* and *A. albopictus* by *Wolbachia* [14], [15].

In summary, the only immune genes up-regulated by *w*MelPop-CLA and *w*Mel in *A. aegypti* are anti-microbial peptides, Toll pathway and melanization genes. However, the same *Wolbachia* strains did not up-regulate these genes in *Drosophila*, and yet dengue interference occurs in this host. This indicates that the up-regulation of these immune effector genes is not required to interfere with dengue virus replication, although it is likely that the immune up-regulation that occurs in mosquitoes, presumably due to the recent association with *Wolbachia*, might enhance this effect.

## Materials and Methods

### Insect rearing

All the mosquito strains used in this study were laboratory lines of *A. aegypti* infected with *w*Mel (MGYP2) or *w*MelPop-CLA (PGYP1), and their tetracycline-treated uninfected counterparts, MGYP2.tet and PGYP1.tet [7], [8]. Adult mosquitoes were kept on 10% sucrose solution at 25°C and 60% humidity with a 12-h light/dark cycle. Larvae were maintained with fish food pellets (Tetramin, Tetra).

The fly experiments were performed with *w^1118^* fly lines stably infected with *w*Mel (*w^1118^w*Mel) [31] and *w*MelPop (*w^1118^w*MelPop) [18] compared to the tetracycline-cured lines derived by the addition of tetracycline (0.3 mg/ml) to the adult diet for two generations. Those lines were confirmed to be free of *Wolbachia* by PCR, using primers specific for the *w*Mel and *w*MelPop IS5 repeat [22]. Females were kept under controlled conditions, low-density (30 females per vial), at 25°C with 60% relative humidity and a 12-h light/dark cycle.

### Sample collection and hybridization

Three replicate pools of 20 female mosquitoes, 8 days post-eclosion were collected from each of the four lines (PGYP1, MGYP2, PGYP1.tet and MGYP2.tet), snap frozen in liquid nitrogen and extracted for total RNA using Trizol (Invitrogen). RNA was then purified using RNeasy kits (Qiagen) according to manufacturer's instructions. Whole-genome microarrays were then used to compare gene expression in the *Wolbachia*-infected lines relative to uninfected controls, using a dual-color reference design. All sample preparations and hybridizations were then carried out by the IMB Microarray Facility at the University of Queensland. Briefly, sample quality was examined using the Agilent 2100 Bioanalyzer (Agilent Technologies) and fluorescent cDNA was synthesized using Agilent Low RNA Input Linear Amplification Kit with Cy3 or Cy5. Each infected line and respective paired tetracycline-treated line was represented by 3 biological replicates (3 pools above). A total of 6 hybridizations were then carried out for each biological replicate, 3 labeled with cy3 and three with cy5 (dye swaps).

### Microarray design

Microarrays were of the 4×44 K format (Agilent) each containing standard control features and 3 technical replicates of each 60 mer feature randomly distributed across the layout. The *A. aegypti* genomic sequence (Vectorbase genome build 1.1) was used for construction of oligonucleotide microarrays using eArray Version 5.0 (Agilent Technologies). After removing probes that cross hybridized, a total of 12,336 transcripts that represented 12,270 genes were spotted onto each microarray [32].

### Microarray data analyses

For each transcript, raw data was extracted and analyzed using Genespring v.9.0 (Agilent Technologies). An intensity dependent (Lowess) normalization (Per Spot and Per Chip) was used to correct for non-linear rates of dye incorporation as well as irregularities in the relative fluorescence intensity between the dyes. Hybridizations from each mosquito line were used as replicate data to test for significance of expression changes using the cross-gene error model. The occurrence of false positives was corrected using the q-value [33], [34]. All array data have been deposited in ArrayExpress (http://www.ebi.ac.uk/microarray-as/ae/) under the accession number E-MEXP-2931.

Functional annotations of *A. aegypti* genes were retrieved from Biomart [35] in Vectorbase [36] and analyzed using the Ontologizer software with the parent child intersection method [37], [38]. The over-expression of particular GO categories in the microarray data set was tested against the distribution of GO categories for the *A. aegypti* genome.

### Virus injection

Dengue virus serotype 2 (DENV-2), strains 92T [9] and ET300 were isolated from human serum collected from patients from Townsville, Australia, in 1992 and East Timor in 2000, respectively. DENV-2 (strains 92T and ET300) was propagated and quantified as described by Frentiu et al [29]. For virus injection, 8 day old *D. melanogaster* females (*w^1118^w*Mel and *w^1118^*tet) and *A. aegypti* females (MGYP2 and MGYP2tet) were briefly anesthetized with CO_2_ and injected under a dissecting scope into their thorax with a pulled glass capillary and a handheld microinjector (Nanoject II, Drummond Sci.). 69 µl of virus stock (10^7^ pfu/ml) or sterile PBS 1X were injected. After injection flies and mosquitoes were maintained under identical controlled conditions, low-density (10 females per vial or cup), at 25°C with 60% relative humidity and 12-h light/dark cycle. Insects were collected 8 days post-injection and kept at −80°C for RNA extraction.

### Quantitative DENV-2 PCR analysis

RNA extraction was done on 15 individual 16 day old females per condition using Trizol (Invitrogen). 1 µg of total RNA was kept to quantify DENV-2 while the rest was used for immune gene expression analysis as described below.

Accumulation of genomic (+RNA) RNA strands was assessed by quantitative real time PCR using hydrolysis probes specific to the 3′ UTR region of the four dengue serotypes [39] with modifications (A.T. Pyke, unpublished data). The sequences of the primers were FWD: 5′-AAGGACTAGAGGTTAGAGGAGACCC-3′ and RWD: 5′-CGTTCTGTGCCTGGAATGATG-3′ and the sequence of the probe was 5′- AACAGCATATTGACGCTGGGAGAGACCAGA-3′. 1 µg of total RNA for each sample was mixed with 0.625 µM of the reverse primer plus 0.2 mM dNTPs. Samples were incubated at 86°C for 15 minutes and 5 minutes on ice, then 5X first strand buffer and 100 U of Superscript III (Invitrogen) was added to a total volume of 20 µl. Samples were incubated at 25°C for 10 minutes, followed by 42°C for 50 minutes and 10 minutes at 95°C to inactivate the transcriptase.

The qPCR reaction consisted of 2 µl of the synthesized cDNAs, 5 µl of 2X LightCycler 480 Probes Master (Roche), 0.5 µM of each primer (see above) and 0.5 µM of the probe (see above) in 10 µl total volume. Reactions were performed in duplicate in a LightCycler 480 Instrument (Roche) with the following conditions: 95°C for 5 minutes, and 45 cycles of 95°C for 10 s, 60°C for 15 s, 72°C for 1 s. A standard curve was created by cloning the DENV-2 3′UTR region fragment into pGEM® T-Easy (Promega). After linearization with Pst I the plasmid was serially diluted into known concentrations and run in parallel, in order to determine the absolute number of DENV-2 copies in each 1 µg of total RNA. First, percentages of individuals infected with dengue were calculated for each treatment. Then only individuals with dengue infection (non zero quantification) were used to examine the effect of *w*Mel on dengue titer using Mann-Whitney *U* tests (Graph Pad Prism 5).

### Quantitative PCR analysis of immune genes

RNA extraction from flies was done using between 10 to 15 individual 8 day old females per condition using Trizol reagent (Invitrogen). To eliminate any contamination by DNA, samples were treated with DNase I recombinant (Roche), in accordance with the manufacturer's instructions. cDNAs were synthesized from 1 µg of total RNA, using oligodT primers and the SuperScript III enzyme (Invitrogen), in accordance with manufacturer's instructions. For each sample qRT-PCR was performed in triplicate on a 10 times dilution of the cDNAs using Platinum SYBR Green (Invitrogen) according to the manufacturer's protocol. Primers are listed in Table S4. The temperature profile of the qPCR was 50°C for 2 minutes (UDG incubation), 95°C for 2 minutes, 45 cycles of 95°C for 5 s, 60°C for 5 s, 72°C for 10 s with fluorescence acquisition of 78°C at the end of each cycle, then a melting curve analysis after the final cycle. The housekeeping gene *rpS17* was used to normalize expression. Target gene to housekeeping gene ratios were obtained for each biological replicate using Q-Gene software [40]. Raw data were graphed as median ± interquartile range (IQR) and outliers beyond 1.5 IQR excluded. Treatment effects on expression ratios were then examined using the Mann-Whitney *U* tests (Graph Pad Prism 5). The occurrence of false positives was corrected using the q-value [33], [34].

## Supporting Information

Figure S1 Immune gene expression in *Drosophila melanogaster* in response to *w*Mel and DENV-2. The expression of immune genes was analyzed by qRT-PCR on individual females injected either with DENV-2 strain 92T (*w^1118^w*Mel D+, *w^1118^*tet D+) or PBS (*w^1118^w*Mel PBS, *w^1118^*tet PBS) in presence/absence of *Wolbachia* strain *w*Mel. Flies were collected 8 days post-injection. Graphs show the target gene to house-keeping gene expression ratio (n = 15, Mann-Whitney *U* test with q-value adjustment, *: q<0.05, **: q<0.01, ***<0.001).
(TIF)

Figure S2 Correlation analysis between dengue titer and immune gene expression in *Drosophila melanogaster* in presence/absence of *Wolbachia* strain *w*Mel (*w^1118^w*Mel, *w^1118^*tet). The values were compared using Spearman correlation coefficients.
(TIF)

Table S1 *Aedes aegypti* transcriptional responses common to *w*Mel and *w*MelPop-CLA infections. Transcripts are ranked by the magnitude of Absolute Fold Change (AFC). Transcript identifiers (Transcript ID) and Description were compiled from Vectorbase. *Drosophila melanogaster* Gene Identifier (Dm Gene ID) and Symbol were compiled from Flybase.
(XLS)

Table S2 *Aedes aegypti* transcriptional responses to *w*MelPop-CLA infection. Transcripts are ranked by the magnitude of Absolute Fold Change (AFC). Transcript identifiers (Transcript ID) and Description were compiled from Vectorbase. *Drosophila melanogaster* Gene Identifier (Dm Gene ID) and Symbol were compiled from Flybase.
(XLS)

Table S3 *Aedes aegypti* transcriptional responses to *w*Mel infection. Transcripts are ranked by the magnitude of Absolute Fold Change (AFC). Transcript identifiers (Transcript ID) and Description were compiled from Vectorbase. *Drosophila melanogaster* Gene Identifier (Dm Gene ID) and Symbol were compiled from Flybase.
(XLS)

Table S4 Oligonucleotide primers used in Real-time qPCR experiments.
(DOC)
