# Supplementary figures and images for: The Relative Importance of Innate Immune Priming in Wolbachia-Mediated Dengue Interference
Source: PLoS Pathog. 2012 Feb 23;8(2):e1002548. doi: 10.1371/journal.ppat.1002548 (PMC3285598; doi:10.1371/journal.ppat.1002548)

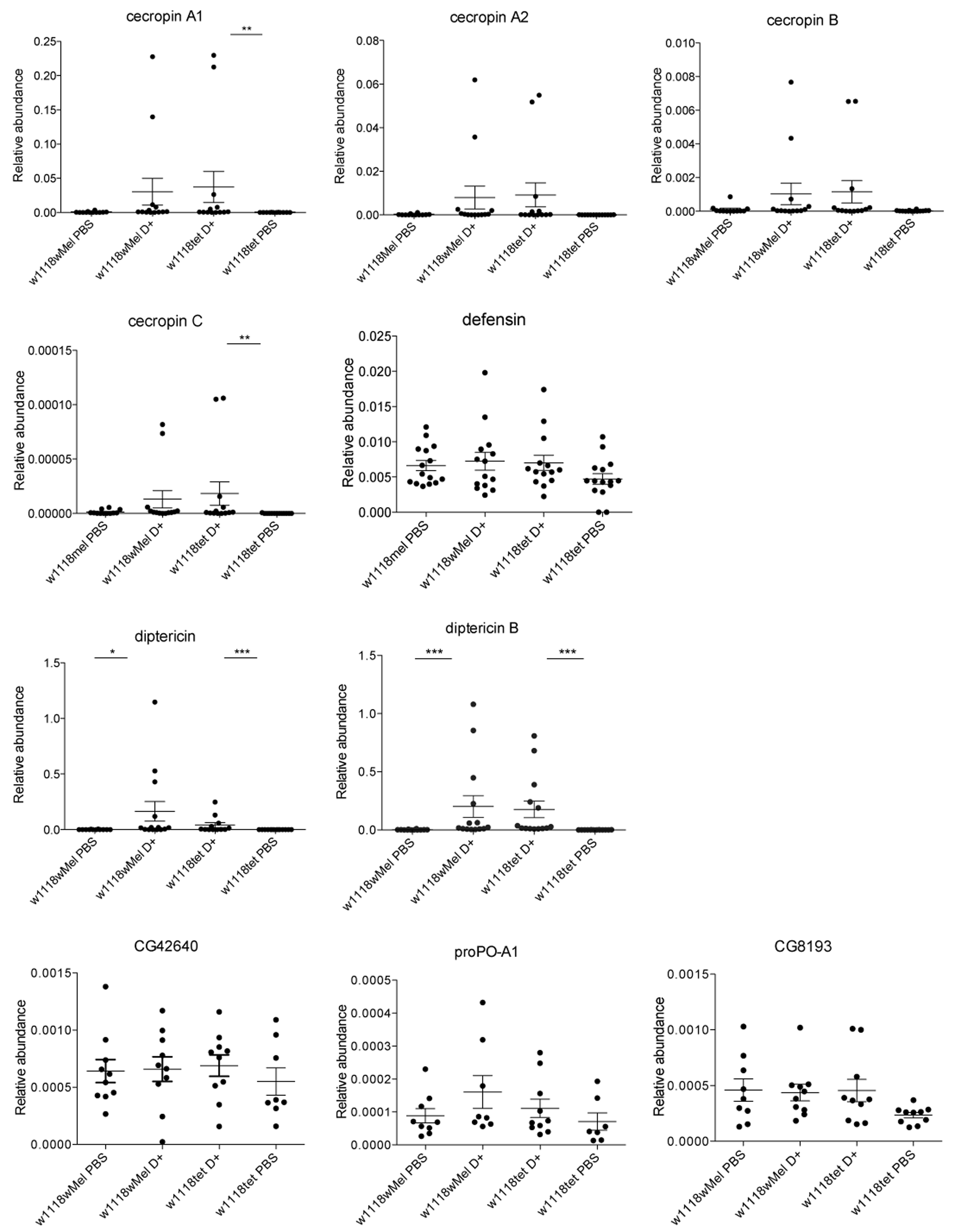

Supplement: Figure S1 — Immune gene expression in Drosophila melanogaster in response to wMel and DENV-2. The expression of immune genes was analyzed by qRT-PCR on individual females injected either with DENV-2 strain 92T (w1118wMel D+, w1118tet D+) or PBS (w1118wMel PBS, w1118tet PBS) in presence/absence of Wolbachia strain wMel. Flies were collected 8 days post-injection. Graphs show the target gene to house-keeping gene expression ratio (n = 15, Mann-Whitney U test with q-value adjustment, *: q<0.05, **: q<0.01, ***<0.001). (TIF) [file ppat.1002548.s001.tif]

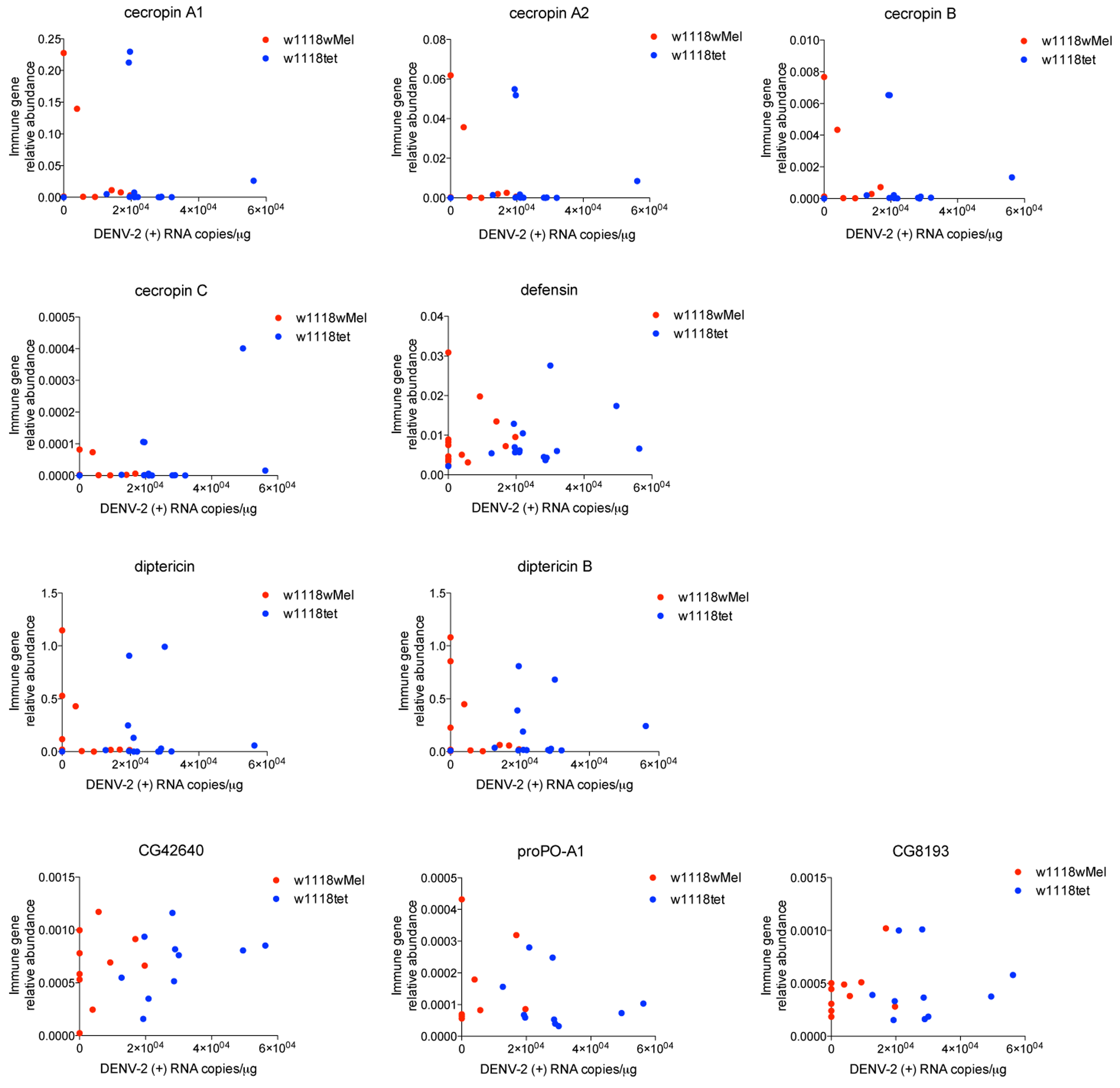

Supplement: Figure S2 — Correlation analysis between dengue titer and immune gene expression in Drosophila melanogaster in presence/absence of Wolbachia strain wMel (w1118wMel, w1118tet). The values were compared using Spearman correlation coefficients. (TIF) [file ppat.1002548.s002.tif]
