# Supplementary material for: The Relative Importance of Innate Immune Priming in Wolbachia-Mediated Dengue Interference
Source: PLoS Pathog. 2012 Feb 23;8(2):e1002548. doi: 10.1371/journal.ppat.1002548 (PMC3285598; doi:10.1371/journal.ppat.1002548)
Supplement: Table S4 — Oligonucleotide primers used in Real-time qPCR experiments. (DOC) [file ppat.1002548.s006.doc]

**Table S4. Oligonucleotide primers used in Real-time qPCR experiments.**

| **Gene name** | **Accession no.** | **Forward Primer 5’-3’** | **Reverse Primer 5’-3’** |
| --- | --- | --- | --- |
| ***A. aegypti*** | | | |
| CecD | AAEL000598 | GCTGTTCGCAATTGTGCTGTT | CAATTTCTTTCCCAGCTTCTTCA |
| DefC | AAEL003832 | GCTGAGTGGGTTCGGTGTAG | CGCGTTACAATAGCCTCCTC |
| Tsf1 | AAEL015458 | TCAGGATCTGATGGCCAAAC | GCCTTGACCTTCTCCAGACA |
| Rps17 | AAEL004175 | CACTCCCAGGTCCGTGGTAT | GGACACTTCCGGCACGTAGT |
| ***D. melanogaster*** | | | |
| CecA1 | FBgn0000276 | GCTGGGTGGCTGAAGAAAAT | CGGCTTGTTGAGCGATTC |
| CecA2 | FBgn0000277 | GGCAAGAAAATCGAACGTGT | GAGCAGTGGCTGCAACATT |
| CecB | FBgn0000278 | GAACGCATTGGTCAGCATAC | AGCGGTGGCTGCAACATT |
| CecC | FBgn0000279 | CAGCATTGGACAATCGGAAG | TCCCAGTCCTTGAATGGTTG |
| Dpt | FBgn0004240 | ACCGCAGTACCCACTCAATC | GGTCCACACCTTCTGGTGAC |
| DptB | FBgn0034407 | GGACTGGCTTGTGCCTTCT | GAGCATATGCCAGTGGTTCA |
| Def | FBgn0010385 | AGGATCATGTCCTGGTGCAT | GGAGAGTAGGTCGCATGTGG |
| PRGP-SA | FBgn0030310 | CGCTATGTGGTCATCCATCA | TGATGATACGCCTGCATGTT |
| GNBP1 | FBgn0040323 | AGCCTTGGGCCAACTATCAT | AGCCTTGGGCCAACTATCAT |
| proPO-A1 | FBgn0261362 | CCTACTGGCAGAAGTCGAGTG | GGTGAACGAGGCGAATATGT |
| CG8193 | FBgn0033367 | GCTACGTGCGCATCTTCAT | CTTGTCCAGCTCGATCATCA |
| CG42640 | FBgn0261363 | ACTCCACGGAGTCGAGTGTC | GAAGTTCTGCAGCTCCTCCA |
| yellow-f | FBgn0041710 | GCACGTTCTGATCATGGAGA | GTCCGCGGATCGTATTTATG |
| Tsf1 | FBgn0022355 | ATGAGAATTGCGAACGATCC | CTGGATGAGACACGACAAGC |
| Fer2lch | FBgn0015221 | GCCCACTACTTCGAGGAGAA | GGGACCTTCATCAGCTTGG |
| Rps17 | FBgn0005533 | CACTCCCAGGTGCGTGGTAT | GGAGACGGCCGGGACGTAGT |
